# Supplementary material for: Identification of photoperiod-induced specific miRNAs in the adrenal glands of Sunite sheep (Ovis aries)
Source: Front Vet Sci. 2022 Jul 22;9:888207. doi: 10.3389/fvets.2022.888207 (PMC9354845; doi:10.3389/fvets.2022.888207)
Supplement: Supplementary file 1 [file Data_Sheet_1.ZIP › Supplementary file/miRDeep2 file.zip!/miRDeep2 file/Novel_Structure/SPLP42A1/Novel_401.pdf]

Provisional ID	:	NC_019459.2_4126
Score total	:	2
Score for star read(s)	:	-1.3
Score for read counts	:	0
Score for mfe	:	1.7
Score for randfold	:	1.6
Score for cons. seed	:	
Total read count	:	167
Mature read count	:	133
Loop read count	:	0
Star read count	:	34

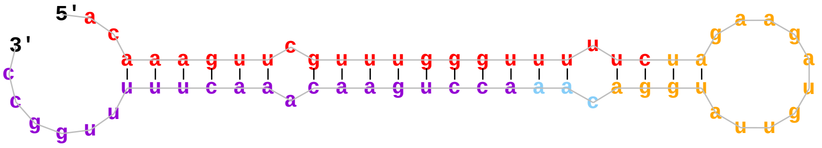

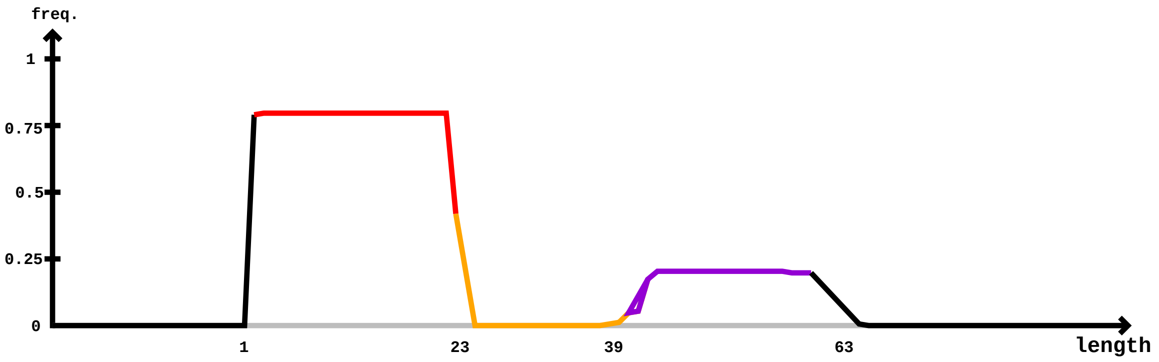


```
                          Mature                                   Star
5'- auaugguauuaaauuggccacaaaguucguuuggguuuuucuagaagauguuauggacaaaccugaacaaacuuuuuggccagcccaauaccauuaggugccaucuacua  -3'   obs
    auaugguauuaaauuggccacaaaguucguuuggguuuuucuagaagauguuauggacaaaccugaacaaacuuuuuggccagcccaauaccauuaggugccaucuacua         exp
    ..((((((((...(((((((.((((((.((((((((((.((((..........)))).)))))))))).)))))).)))))))...))))))))(((((...)))))...   reads   mm   sample
    ...................aAaaaguucguuuggguuuuu......................................................................   62      1    seq
    ...................aAaaaguucguuuggguuuuuc.....................................................................   66      1    seq
    ...................aAaaaguucguuuggguuuuucu....................................................................   4       1    seq
    ....................caaaguucguuuggguuuuu......................................................................   1       0    seq
    ........................................................aAaaaccugaacaaacuuu...................................   1       1    seq
    .........................................................Aaaaccugaacaaacuuuuugg...............................   1       1    seq
    ..........................................................aaaAcugaacaaacuuuuuggc..............................   1       1    seq
    ..........................................................aaaccugaacaaacuuuuuggc..............................   3       0    seq
    ..........................................................aaaUcugaacaaacuuuuuggcc.............................   1       1    seq
    ..........................................................aaaccugaacaaacuuuuuggcA.............................   1       1    seq
    ...........................................................aaUcugaacaaacuuuuuggc..............................   1       1    seq
    ............................................................accugaacaaacuuuuuggcA.............................   1       1    seq
    ............................................................aUcugaacaaacuuuuuggcc.............................   18      1    seq
    ............................................................aUcugaacaaacuuuuuggccag...........................   1       1    seq
    .............................................................ccugaacaaacuuuuuggcU.............................   1       1    seq
    .............................................................Ucugaacaaacuuuuuggcca............................   4       1    seq
```
